# Pregnancy Disorders: A Potential Role for Mitochondrial Altered Homeostasis

**DOI:** 10.3390/antiox13080979

**Published:** 2024-08-13

**Authors:** Juan M. Toledano, María Puche-Juarez, Jose Maria Galvez-Navas, Jorge Moreno-Fernandez, Javier Diaz-Castro, Julio J. Ochoa

**Affiliations:** 1 Department of Physiology, Faculty of Pharmacy, Campus Universitario de Cartuja, University of Granada, 18071 Granada, Spain; jmtoledano@ugr.es (J.M.T.); javierdc@ugr.es (J.D.-C.); jjoh@ugr.es (J.J.O.); 2 Institute of Nutrition and Food Technology “José Mataix Verdú”, University of Granada, 18071 Granada, Spain; 3 Nutrition and Food Sciences Ph.D. Program, University of Granada, 18071 Granada, Spain; 4 Centro de Investigación Biomédica en Red de Epidemiología y Salud Pública (CIBERESP), 28029 Madrid, Spain; jmaria.galvez.easp@juntadeandalucia.es; 5 Cáncer Registry of Granada, Andalusian School of Public Health, Cuesta del Observatorio 4, Campus Universitario de Cartuja, 18011 Granada, Spain; 6 Department of Biochemistry and Molecular Biology II, Faculty of Pharmacy, Campus Universitario de Cartuja, University of Granada, 18071 Granada, Spain; 7 Instituto de Investigación Biosanitaria (IBS), 18016 Granada, Spain

**Keywords:** mitochondria, gestation, pregnancy complications, preeclampsia, fetal growth restriction, gestational diabetes mellitus, bioenergetics, oxidative phosphorylation, mitochondrial dynamics

## Abstract

Pregnancy is a complex and challenging process associated with physiological changes whose objective is to adapt the maternal organism to the increasing energetic requirements due to embryo and fetal development. A failed adaptation to these demands may lead to pregnancy complications that threaten the health of both mothers and their offspring. Since mitochondria are the main organelle responsible for energy generation in the form of ATP, the adequate state of these organelles seems crucial for proper pregnancy development and healthy pregnancy outcomes. The homeostasis of these organelles depends on several aspects, including their content, biogenesis, energy production, oxidative stress, dynamics, and signaling functions, such as apoptosis, which can be modified in relation to diseases during pregnancy. The etiology of pregnancy disorders like preeclampsia, fetal growth restriction, and gestational diabetes mellitus is not yet well understood. Nevertheless, insufficient placental perfusion and oxygen transfer are characteristic of many of them, being associated with alterations in the previously cited different aspects of mitochondrial homeostasis. Therefore, and due to the capacity of these multifactorial organelles to respond to physiological and pathophysiological stimuli, it is of great importance to gather the currently available scientific information regarding the relationship between main pregnancy complications and mitochondrial alterations. According to this, the present review is intended to show clear insight into the possible implications of mitochondria in these disorders, thus providing relevant information for further investigation in relation to the investigation and management of pregnancy diseases.

## 1. Introduction

Mitochondria are cellular organelles derived from the evolution of ancient eukaryotic cells that established an endosymbiotic relationship with aerobic bacteria. Consequently, and even though most of their original genome has colonized the host genome over millions of years, some genes have been conserved within the matrix of the organelle, leaving a circular genome called mitochondrial DNA (mtDNA). It works in conjunction with the nuclear genome and other organelles to carry out the large number of functions in which mitochondria are involved. Among them, energy production in the form of ATP must be highlighted, which is tightly associated with the generation of potentially damaging reactive oxygen species (ROS). Mitochondria generate a dynamic network, which is subject to change and reshaping in response to membrane potential and cell metabolism and is also related to apoptosis pathways [1]. Due to their functions, mitochondria are considered key organelles to maintain the proper activity of tissues and organs with high energy requirements like the placenta, a structure of great importance for fetal development. It acts as an active interface between maternal and fetal blood and is responsible for a wide range of fundamental physiological functions for pregnancy maintenance, such as providing a physical and immune barrier, mediating the transfer of gases, nutrients, and wastes, and producing/secreting several hormones, cytokines, and signaling molecules [2].

Mitochondrial impairments are believed to be associated with a number of health disorders associated with oxidative stress, including type 2 diabetes, cardiovascular diseases, or neurodegenerative illnesses. Partial blood flow occlusion and local hypoxia are common characteristics in several diseases, which would in turn have repercussions on mitochondria. Due to their oxygen consumption in order to produce ATP, modification in oxygen availability can lead to increased ROS production, and this excessive oxidative stress can lead to mitochondrial damage, thus affecting their structure and function [3]. When it comes to pregnancy, several pregnancy complications are associated with augmented oxidative stress, including preeclampsia (PE), fetal growth restriction (FGR), and gestational diabetes mellitus (GDM) [4]. Indeed, it has been observed that pregnant women with mitochondrial alterations are more likely to undergo these disorders and also have a higher severity in their symptoms [5]. These pathologies constitute the leading causes of maternal/fetal morbimortality and are frequently related to poor placental perfusion, reduced oxygen supply, and/or exposure to several insults like high levels of glucose, fatty acids, and inflammatory mediators, which can in turn lead to dampened function of the placenta [6,7,8]. Since these detrimental changes can also modify the structure and functionality of mitochondria, these organelles may represent a possible explanation for the etiology of pregnancy complications [9]. According to all this, the present review is intended to summarize the main evidence on the relationship between major pregnancy disorders (preeclampsia, fetal growth restriction, and gestational diabetes mellitus) and mitochondrial alterations, regarding content, structure, mtDNA state, energy production, ROS generation, biogenesis, dynamics, and apoptosis. Providing more comprehensive insight into this topic is key to supporting the search and testing of interventional strategies (e.g., mitochondrial-targeted antioxidants) aimed at ameliorating these complications.

## 2. Mitochondria: Physiological Role and Homeostasis

Mitochondria are multifunctional organelles with an exclusively maternal inheritance, which means that diseases related to mitochondrial DNA (mtDNA) mutations will only pass to the progeny from mothers. These organelles perform a number of key cellular functions even though they are more known for being the powerhouses of the cell. Indeed, they require energy production in the form of ATP, which is carried out thanks to a process known as oxidative phosphorylation (OXPHOS). Nevertheless, they are also involved in several physiological processes, such as autophagy, apoptosis, innate immunity, calcium homeostasis, iron metabolism, stem cell reprogramming, and redox signaling [10].

The ultrastructure of these organelles is of great importance in order to utterly understand their functions and the different processes they experiment. In this sense, the main feature of mitochondrial ultrastructure is their double membrane system, composed of an outer mitochondrial membrane (OMM) facing the cytosol and an inner mitochondrial membrane (IMM), in contact with the intern matrix of the organelle where the mitochondrial genome is localized. Between both membranes, there is a compartment called the intermembrane space (IMS), which is quite relevant for membrane potential maintenance and, therefore, ATP production [11]. The aforementioned genome, also called mitochondrial DNA (mtDNA), contains 13 genes encoding key proteins for the OXPHOS process, as well as 2 genes encoding ribosomal RNAs, and 22 genes encoding transfer RNAs, which are required for the translation of the protein-encoding genes. Therefore, the mitochondrial genome is composed of 37 genes that are fundamental for proper OXPHOS activity [12].

ATP can be generated through the oxidation of different biomolecules, like sugars, fats, and proteins, acting as the fuel that allows for key cellular processes, including molecule synthesis and active transport. The high-energy molecules NADH and FADH2 produced after processes like the tricarboxylic acid cycle (also called the citric acid cycle or Krebs cycle) or β-oxidation provide the electrons required for the functioning of the electron transport chain [13]. This chain is composed of five protein complexes located in the IMM and is responsible for creating the membrane potential required for ATP synthesis. In this last step of OXPHOS, complex V (also known as ATP synthase) couples ADP phosphorylation with the dissipation of the proton gradient previously obtained. Alternatively, protons can re-enter the mitochondrial matrix through uncoupling proteins (UCPs), which dissipate the proton gradient preventing them from being harnessed in ATP generation since their energy is used for thermogenesis. As a result, they uncouple substrate oxidation from ATP production while reducing ROS generation [13].

On a regular basis, electron leakage takes place during electron transport in OXPHOS, producing oxygen-reactive species (ROS) as byproducts. As a result, this process is the major source of ROS generation in cells, including placental trophoblasts. However, OXPHOS impairments can lead to excessive oxidative stress, affecting mitochondrial respiration efficacy, damaging cellular biomolecules (such as lipids, proteins, and nucleic acids), and triggering cell death pathways [10,14]. The superoxide radicals produced in oxidative respiration can be eliminated by superoxide dismutase (SOD), converting them into H_2_O_2_, which is the substrate for a cytosolic reduction reaction carried out by catalase [15]. H_2_O_2_ can also be reduced and transformed into water and by peroxidase or peroxiredoxin, which, together with glutathione and thioredoxin systems, support the defense against oxidative stress-related damage to cellular components [16]. As the main ROS generator, mitochondria are also vulnerable to damage mediated by these, including the alteration of mtDNA and lipid peroxidation, which in turn may disrupt electron transport chain complex expression and membrane integrity, respectively [16].

After they are generated, mitochondria are far from being static organelles since they undergo several remodeling processes that include fusion (the combination of two mitochondria to produce a bigger one), fission (the fragmentation of a mitochondrion to produce two smaller ones), mitophagy (the elimination of damaged/dysfunctional mitochondria through an autophagy process), and motility (the transport of the organelles along the cytoplasm). The combination of these four processes is referred to as “mitochondrial dynamics”, and together with mitochondrial biogenesis (the generation of new organelles), it is key to the maintenance of a healthy and functional mitochondrial network within cells [17]. Broadly speaking, biogenesis is regulated by the peroxisome proliferator-activated receptor-γ coactivator-1α (PGC-1α), which promotes the downstream activity of nuclear respiratory factors 1 and 2 (NRF1 and NRF2), which ultimately control mitochondria-related gene expression through the mitochondrial transcription factor A (TFAM) [18]. Fusion is mediated through the activity of mitofusins 1 and 2 (MFN1 and MFN2) and optic atrophy 1 (OPA1) protein, which carry out the merging of OMM and IMM, respectively. OPA 1 is also fundamental for the maintenance of cristae integrity [12,19]. Fission is allowed by the dynamin-related protein 1 (DRP1), which is more associated with membrane constriction, and dynamin 2 (DNM2), which is more related to final scission, even though mitochondrial fission 1 protein (FIS1) also has a role in the process, recruiting DRP1 to the site of constriction [12]. As for mitophagy, it can be developed through two major pathways. The first one is mediated by the axis formed by PTEN-induced kinase 1 (PINK1) and E3 ubiquitin-protein ligase parkin (Parkin), whose activation allows the recruitment of autophagy receptors such as calcium binding, coiled-coil domain 2 (CALCOCO2/NDP52), and optineurin (OPTN). The second one is a receptor-mediated pathway, which is triggered by several mitochondrial receptors, like FUN14 domain containing 1 (FUNDC1), BCL2 interacting protein 3 (BNIP3), BCL2-like 13 apoptosis facilitator (BCL2L13), FKBP prolyl isomerase 8 (FKBP8), and prohibitin 2 (PHB2) [20].

Finally, as key signaling organelles essential for the metabolic function of cells, mitochondria also have a role in the regulation of apoptosis, a process that enables the removal of impaired cells to keep tissues healthy and functional. Apoptosis is triggered by either extracellular or intracellular signals, leading to caspase activation and subsequent downstream cleavage cascades that result in cell elimination. Increased ROS production and oxidative stress can lead to alterations in Ca^2+^ homeostasis (mainly an increased Ca^2+^ efflux), which in turn opens mitochondrial permeability transition pores (mPTP) and leads to cell death [16]. ROS-associated impairments in mitochondrial integrity can result in augmented release of cytochrome c to the cytosol due to increased OMM permeability mediated by pro-apoptotic BCL-2 family proteins. Once out of the mitochondria, cytochrome c leads to the generation of complexes called apoptosomes, which activate caspase 9 and enable the cascade responsible for the execution of apoptosis events. The increased permeability and pore establishment are frequently reflected in a swelling of the mitochondrial matrix, disorganization of their structure, and, eventually, organelle rupture [10].

With regard to mitochondrial morphology in normal placentas, it is important to consider the differences in these organelles between cytotrophoblast and syncytiotrophoblast cells. The first ones are large and round and have lamellar cristae with an orthodox configuration. On the contrary, those in syncytiotrophoblasts are smaller and irregular, showing protuberances in the OMM and IMM, a condensed matrix, and cristae with vesicular regions connected by narrow tubules [2]. Finally, the relevance of placental mitochondria in steroidogenesis deserves to be highlighted. Differentiation into syncytiotrophoblasts is accompanied, among other changes, by the acquisition of the steroidogenic machinery consisting of an electron transport chain composed of cytochrome P450scc CYP11A1, which receives electrons provided by NADPH+H^+^ through adrenodoxin and adrenodoxin reductase. All these proteins are in the IMM, allowing the transformation of cholesterol into pregnenolone. An additional enzyme also located in the IMM finally transforms pregnenolone into progesterone, a key steroid hormone in pregnancy maintenance and especially in the prevention of fetal rejection [2,21].

All these important mitochondria-related processes have been reported to be disrupted in one way or another during different pregnancy complications. This will be discussed in the following sections, showing the most relevant and recent findings regarding the relationship between mitochondrial alterations and pregnancy-related disorders. 

## 3. Materials and Methods

The bibliographic research performed from January 2024 to March 2024 utilized the main biomedical databases and sources, including Medline (via PubMed), the Cochrane Library, Elsevier, and Dialnet, limiting the search to the last 10 years. Among the articles found, only those recent publications addressing the subject of this narrative review (the relationship between pregnancy complications and mitochondrial alterations) were included. Only articles in English have been accepted from the search since it is the lingua franca of science. As for the keywords applied, these included mitochondria, gestation, pregnancy complications, preeclampsia, fetal growth restriction, gestational diabetes mellitus, bioenergetics, oxidative phosphorylation, mitochondrial dynamics. The use of medical subject heading (MSH) was also taken into consideration in those words possibly leading to a misunderstanding in the browser. In addition, the boolean operators “AND” and “OR” were combined with keywords in order to find more pertinent articles. In this sense, “AND” was used between terms to increase both the sensitivity and specificity of the search process (example: mitochondria dynamics AND pregnancy complications); “OR” was applied to link synonyms (example: mitochondria AND (pregnancy complications OR preeclampsia OR fetal growth restriction OR gestational diabetes mellitus)).

**Figure 1:**
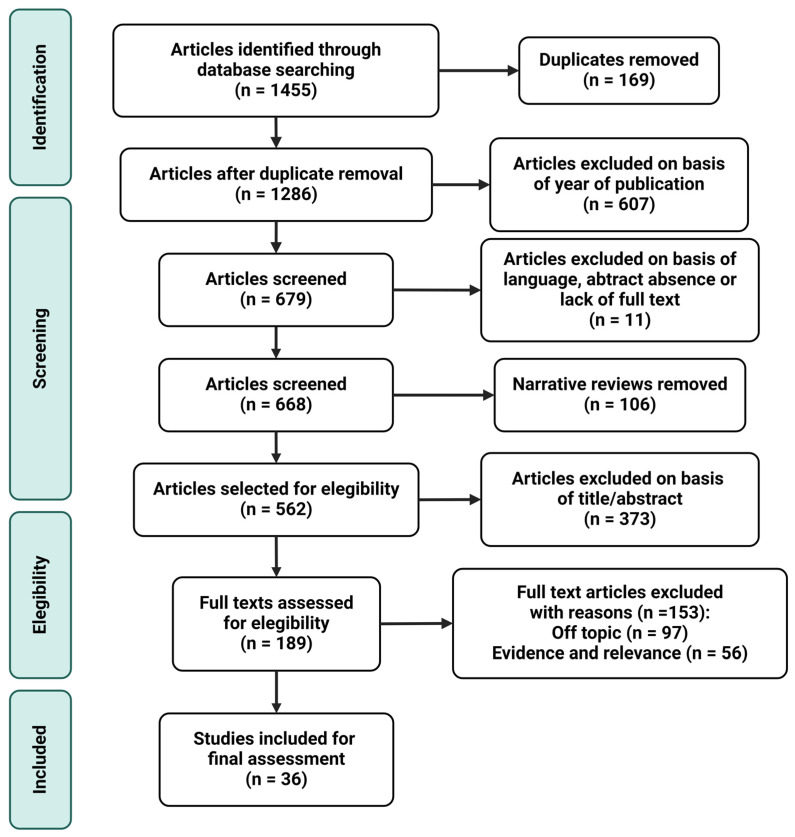
Manuscript selection flowchart.

The inclusion criteria considered were the following: controlled trials, observational studies, animal models, in vitro studies, and meta-analysis; publication within the last 10 years; and English language (especially those papers involving pregnancy disorders and mitochondrial affectation). On the other hand, narrative reviews, abstract absence, not full text, publication before the last 10 years, and not-English language were used as exclusion criteria. Proper article organization, citation, and bibliography management were assured by using EndNote X8.0.2 as the reference software. Figure 1 shows the search methodology and the process carried out to select the articles finally included in the narrative review.

## 4. Results and Discussion

### 4.1. Mitochondria and Preeclampsia

Preeclampsia (PE) is a disorder affecting 3–7% of pregnancies, related to maternal endothelial dysfunction, and is considered one of the most serious and life-threatening pregnancy complications [22]. The disease progresses in two stages: firstly, an abnormal placentation in the first trimester, which is followed by a syndrome taking place in the later second and third trimesters, related to higher circulating levels of anti-angiogenic factors like soluble Flt-1 (sFlt-1) and soluble endoglin (sENG) [23]. Depending on when the syndrome takes place, this condition can manifest with an early-onset PE (before 34 weeks) or a late-onset PE (after 34 weeks) [24]. Its most common clinical features are high blood pressure (systolic blood pressure over 140 mm Hg and/or diastolic blood pressure over 90 mm Hg), proteinuria, and damage in several organs, including the kidney, liver, and central nervous system, together with pulmonary edema and a drop in platelet count. The lack of early management can lead to more severe consequences in these organs [22,24]. Even though the mechanism related to abnormal placentation is controversial, it is known that uteroplacental ischemia and hypoxia are responsible for the hypertensive and multi-organ failure response that characterizes this syndrome in its second stage [22]. This ischemia is a consequence of a shallow trophoblast invasion that fails to remodel uterine spiral arteries and augments placental vascular resistance, leading to difficulties in organ perfusion. Hypoxia would also be the cause of increased oxidative stress, as well as endothelial dysfunction that takes place in an sFlt-1- and sENG-independent manner [25]. Many possibilities have been proposed for the dysfunction observed in the placenta during the first stage, including augmented oxidative stress (either due to increased ROS production or decreased antioxidant capacity in the placenta), altered activity of natural killer (NK) cells, and even genetic and environmental factors. Nevertheless, the dysfunctional placenta produces the release of several soluble toxic factors in the maternal bloodstream, resulting in inflammation, endothelial dysfunction, and systemic failure [22,23]. This complication is also related to FGR and preterm birth (PTB) [24].

Regular, uncomplicated pregnancies undergo an increase in free radical production and subsequently, augmented lipid peroxidation, which is counteracted by an upregulation in antioxidant capacity [26]. However, PE is related to increased lipid peroxidation (among other oxidative stress markers both circulating and in placental tissue) and reduced antioxidant activity at the same time. It must be highlighted that lipid peroxidation is not only influenced by oxidative status but also by lipid levels, and in PE, there is an increase in lipids and modifications in their metabolism with an impact on vascular function [27]. The antioxidant systems that get compromised in preeclamptic pregnancies include superoxide dismutase (SOD), catalase (CAT), and glutathione peroxidase (GPX) [6,28,29], reflecting an imbalance between pro-oxidants and antioxidants mechanisms. The periodic ischemia and reperfusion suffered by the placenta during delivery results in oxidative stress, which induces cytokine release into the maternal circulation, possibly leading to inflammatory responses [25]. These oxidative stress markers are also more elevated in PE pregnancies compared to normal pregnancies [30]. On the other hand, a significant source of oxidative stress is associated with the hyperactivity of xanthine oxidase, consequently leading to an increase in serum uric acid. Indeed, mitochondrial dAMP and xanthine oxidase have an important synergistic effect in hypertensive disorders of pregnancy, being a useful marker for hyperuricemia due to an increased xanthine oxidase activity (and thus ROS production) serum uric acid to serum creatinine ratio [31].

Some studies have suggested that adverse pregnancy outcomes like PE could be prevented by using cytoprotective agents targeting the mitochondria. In this sense, McCarthy et al. [32] exposed human umbilical vein endothelial cells to plasma from women with PE, reporting a decrease in mitochondrial function, as well as augmented mitochondrial superoxide production. However, the pre-treatment of cells with the mitochondrial-targeted antioxidant MitoTEMPO (a mitochondria-targeted SOD mimetic) protected them against cell death induced by peroxide, also normalizing mitochondrial metabolism and reducing mitochondrial superoxide production. On the other hand, the study conducted by Yang et al. [33] found significantly high oxidative stress, mitochondrial damage, and reduced GPX activities in the placentas of subjects with PE. The late administration of MitoQ (lipophilic triphenylphosphonium cation and coenzyme Q10) to these placentas ameliorated uterine perfusion pressure, while early administration proved to be detrimental. In these studies, mitochondrial-targeted antioxidant interventions showed more effectivity than general antioxidant treatment [32,33].

The implications of placental mitochondria in PE have been well assessed and documented. Nevertheless, it is important to highlight that, in some cases, there are inconsistencies among the results obtained with regard to the content, structure, and function of this organelle. In PE pregnancies, changes in mitochondrial fusion/fission balance, mitochondrial content, and placental cell apoptosis have been reported [34]. Proteomic analyses of placentas from PE pregnancies show the relevancy of several functions related to mitochondria, including the citric acid cycle, the electron transport chain, fatty acid oxidation, and Ca^2+^ homeostasis, which might get altered during this condition [35]. In addition, mitochondria of preeclamptic placental tissue have also reported increased swelling and damaged cristae, as well as decreased expression and activity of OXPHOS complexes, including ATP synthase [36]. Among the affected complexes, early-onset PE pregnancies have reported a reduction in the activity of complexes I, III, and IV compared with normotensive controls (with no changes in gene or protein expression). It has been proposed that mitochondrial unfolded protein response (mtUPR), which englobe a number of compensatory processes related to protein homeostasis and antioxidant defenses, may be associated with these results, possibly being a therapeutic target for placental function restoration [37]. A reduced electron flow through complex III might contribute to the excessive production of ROS, which is frequently reported in PE cases [36]. Nevertheless, late-onset PE has reported opposite results, characterized by an increased activity of complexes II and III [38]. Likewise, regarding mitochondrial energy production, some studies have reported reduced expression of electron transport chain complexes and decreased OXPHOS activity [35,36,39], whereas others show the opposite [38]. PE onset, severity, and gestational age have been proposed as possible explanations for this, a priori, contradictory results [40]. Furthermore, metabolomic research related to dysfunctional mitochondria from PE placentas found that 21 metabolic pathways reported a significant downregulation, especially four of them associated with lipid metabolism in general and fatty acid catabolism in particular [41].

As for maternal circulating cell-free mtDNA, it has been related to PE development through a negative correlation since lower levels of this marker have been reported in women experimenting with this complication [42]. However, some studies have also found increased levels of this marker in PE, this material being presumably derived from the dysfunctional placenta [43,44]. Likewise, regarding placental mtDNA content, increased levels have been found in early-onset but not late-onset PE, suggesting different mitochondrial responses depending on the different pathophysiology of the disease. These samples, together with those related to late-onset PE, also reported an increase in citrate synthase activity, which would be indicative of an increased amount of these organelles [34]. Nevertheless, other studies have reported that both mtDNA copy number and citrate synthase activity are reduced in PE samples [45]. These parameters are linked to mitochondrial content within cells and, therefore, to mitochondrial biogenesis processes. In this sense, gene expression of an important biogenesis protein like TFAM seems to get reduced in early-onset PE placental samples compared to a control, with no difference reported for other proteins like NRF1 [34]. PGC1α has also reported reduced protein expression levels in PE pregnancies [40,45], together with sirtuin 3 (SIRT3) [40], supporting the abnormal regulation of mitochondria generation during this common pregnancy complication.

When it comes to mitochondrial dynamics, genes related to this group of processes experiment with several changes in placental samples affected by pregnancy pathologies like PE [46]. Protein expression of several fusion-related proteins has been reported to be impaired in PE-term placentas, including MFNs and OPA1 [40], whereas no differences were reported regarding fission proteins like DRP1 and FIS1 [40,41]. This is supported by other studies showing similar results, which also conclude that defects specifically related to MFN2 could cause mitochondrial alterations that would negatively affect trophoblastic cells’ viability in PE [47]. However, other studies have shown increased expression of fusion markers like MFN1 and MFN2 compared to regular pregnancies [38,40] or even augmented fission represented by higher levels of DRP1 and increased fragmentation of these organelles detected by transmission electron microscopy [48]. Additionally, early-onset PE placentas have also shown increases in the mitochondrial fusion regulator OPA1 in some cases, suggesting a mechanism to protect the mitochondria content of altered trophoblasts [34]. On the other hand, the ratio between the gene expression of TOMM20 (mitochondrial import receptor subunit TOM20 homolog) and Parkin has arisen as a potential marker to discern unhealthy placental tissue with regard to this pathologic condition [46].

Since mitophagy allows for the selective removal of dysfunctional mitochondria to provide cells with organelles suitable for proper energy metabolism, defects in its pathways might be responsible for augmented ROS production and, eventually, the appearance of PE. According to this, several studies have been performed to further explore this potential relationship. BNIP3 expression has been reported as inhibited in PE pregnancies [40,49], leading to a higher accumulation of impaired swollen mitochondria, irrespective of PINK1 levels, which did not change [49]. This has been supported by another study which found reduced BNIP3 gene expression in the decidua of human placentas from both early and late PE [50]. On the contrary, higher protein expression of BNIP3 [45], PINK1, and Parkin [48] has also been reported in PE placentas by different recent research. As for the FUNDC1 receptor, reduced mRNA expression and lower levels of protein ubiquitination were shown in the placental tissue of women suffering from PE [51,52].

Alterations in mitochondrial integrity have also been documented in animal models mimicking PE, showing impairments in the structure of placental mitochondria such as swelling and cristae disappearance [53,54]. In fact, mitochondria swelling is an apoptosis feature that has been reported in PE placentas also from human studies [35], as well as altered levels of many apoptotic proteins [45,55]. On the other hand, PE has been related to impairments in mitochondria-dependent Ca^2+^ signaling, which may lead to apoptosis affectation in placental tissue [56]. Furthermore, and as stated before, increased ceramide levels have also been documented; these are considered inducers of cellular death mechanisms [48].

Placental endothelial cells also seem to be affected by PE since the exposure of these cells to serum from pregnant mothers with PE has reported impairments in mitochondrial structure (damaged cristae, enlarged intramembrane space, and organelle swelling), augmented ROS generation, and reduced viability. There is a similarity of these findings with those observed in FGR, which suggests a common course in the development of both diseases [57].

**Figure 2:**
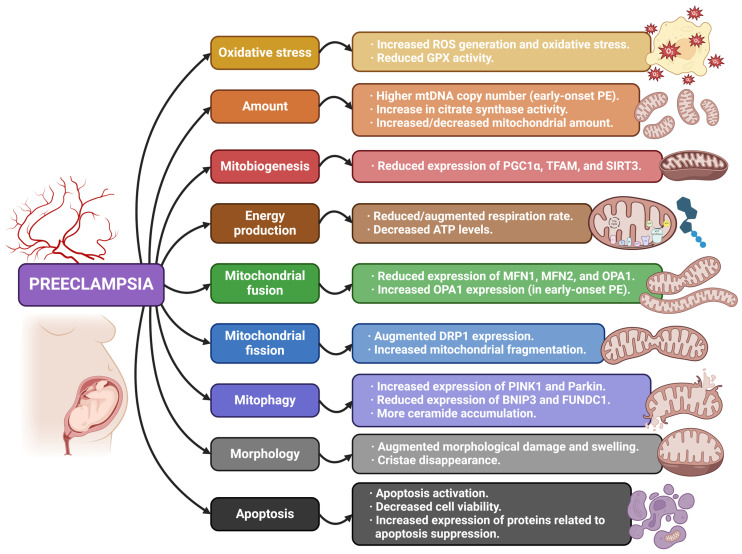
Summary of the mitochondrial alterations associated with preeclampsia found in the reviewed articles.

It is also important to highlight that all these possible mitochondrial alterations and dysfunctions discussed can affect steroidogenesis due to the importance of the organelle in the process. In this sense, abnormal generation of pregnancy-related steroid hormones has been associated with PE, represented by a lower estrone/androstenedione ratio, reduced estradiol and estrone levels, higher concentrations of 20α-dihydroprogesterone (20α-DHP) and 20α-DHP/progesterone ratios, lower pregnenolone sulfate levels, and decreased expression of aromatase [58]. Table 1 and Figure 2 summarize the main information and findings concerning the reviewed articles related to PE.

### 4.2. Mitochondria and Fetal Growth Restriction

Fetal growth restriction (FGR), also known as intrauterine growth restriction (IUGR), takes place when the fetus fails to reach its growth potential and is related to a fetal weight less than the 10th percentile for gestational age. It is a frequent cause of fetal, perinatal, and neonatal morbimortality, associated with a variety of maternal, placental, or fetal risk factors, including smoking, infection, obesity, or malnutrition, even though most cases remain idiopathic. Therefore, any of these elements, either combined or alone, can cause FGR [59,60]. Another pregnancy complication highly related to FGR is a low birth weight (LBW), which is considered as any weight under 2500 g, independent of gestational age. LBW neonates are considerably more likely to undergo health problems, possibly leading to death, compared to regular weight infants. Even though LBW pathophysiology is still unknown, FGR and PTB are frequently considered its main causes [61,62].

With regard to FGR etiology, placental malfunction is the more common feature of this pregnancy complication, resulting in diminished uteroplacental blood flow and, consequently, differences between placental nutrient supply and fetus demand. Organ insufficiency has been proposed to have its origin in early pregnancy during trophoblast invasion of the spiral arteries, this being a process with high energy demand, also generating ROS and resulting in increased oxidative stress. This process requires high energy availability for cell growth, proliferation, and metabolic activity, which generates ROS and oxidative stress. The affectation of spiral artery development leads to ischemia–reperfusion, therefore exacerbating oxidative stress and placental damage [63].

Indeed, lipid peroxidation markers have been reported to be higher in both maternal and umbilical cord plasma, together with placental tissue in pregnancies with FGR, highlighting a relevant role for oxidative stress in this pregnancy complication [64]. Expression of SIRT3, a protein associated with mitochondrial respiration regulation and ROS generation reduction, has been reported to be lower in placentas from FGR pregnancies, which was supported by an increase in oxidative damage markers detected, especially those related to nucleic acids [65,66,67]. However, increased protein levels of SIRT3 have also been reported in FGR placental samples [68]. On the other hand, placentas related to FGR show a bigger presence of aging markers, including telomere shortening and reduced telomerase activity [69,70].

The role that mitochondrial impairment plays in FGR is not clear yet even though there is evidence highlighting that there may be an association between them. During FGR, hypoxic conditions may take place because of the reduction in placental blood flow. This hypoxic stress has been suggested to induce mitochondrial biogenesis and, therefore, an increase in mitochondrial content as a protective response in an attempt to meet the metabolic demands of the tissue through stimulation of its bioenergetic capacity [71,72]. In fact, a significant rise in mtDNA copy number has been found in FGR placentas in comparison to a control [63,65,66,67], sometimes with an increased mutation rate [65], as well as an increase in mitochondrial respiration that has been reported in trophoblasts from FGR placentas. However, the mtDNA copy number was found to be lower when it comes to isolated cytotrophoblast cells [63]. These findings do not seem to be consistent with other studies that reported a reduction in mtDNA and mitochondrial protein content in the placentas from FGR pregnancies, which was correlated with fetal weight and associated with a downregulation of estrogen-related receptor γ (ERRγ) expression. In this sense, mtDNA damage caused by increased placental ROS could lead to an inhibition of adaptive biogenesis and a decrease in mitochondrial respiratory activity [73]. These apparent different outcomes within the same pathology may be related to the timing and/or severity of the insult [63,74]. Consistently with these results, placentas where the mitochondrial amount was reduced showed decreased gene expression of biogenesis regulators like PGC1α and sirtuin 1 (SIRT1) (especially when FGR was combined with PE) [73], whereas those where mtDNA was increased, NRF1 expression was also augmented [63].

On the other hand, FGR seems to be associated with an increased mtDNA copy number measured in maternal blood, whose alterations are related to reductions in the efficiency of the electron transport chain and oxidative phosphorylation, together with augmented ROS generation. Consequently, mtDNA copy number in maternal venous blood has been proposed as a predictor of FGR risk [75]. However, the inverse correlation has also been found regarding umbilical cord blood. On the other hand, high maternal circulating mtDNA levels have been associated with lower oxygen tension in the cord vein [74,75].

When it comes to assessing the expression of the electron transport complexes, a reduction was reported in those placentas from FGR pregnancies. However, these results seem controversial since augmented OXPHOS activity has also been reported. The complexity of placental tissue structure and the different cell types presented in its maternal and fetal sections have been proposed as a potential explanation for these results [63,68]. Additionally, these variations have also been explained as possible consequences of a metabolic compensatory mechanism and modifications regarding other pathways like glycolysis, which is less efficient in energy generation. On the other hand, rat models showed reduced oxidation rates of some molecules like succinate, α-ketoglutarate, and pyruvate, together with augmented manganese SOD concentrations [67,76].

As for mitochondrial dynamics, protein expression of MFN1 has been reported to be reduced in FGR placentas, suggesting a possible impairment in mitochondrial fusion. TOMM20, an importer protein with a key role in mitophagy, has been shown to be augmented in this pregnancy complication, favoring the selective removal of damaged organelles [77]. In addition, the TOMM20/Parkin ratio has also been proposed as a potential marker to discern unhealthy placental tissue in FGR, the same way it has been considered in PE [46]. The potential relationship between FGR and mitophagy has also been addressed in a model in which cadmium exposure was applied as an environmental stressor triggering FGR in mice. The intervention reported an increase in BNIP3-related mitophagy, leading to a higher rate of dysfunctional mitochondria elimination, which has been suggested to be counteracted by the administration of melatonin [78,79].

Placental endothelial cells also appear to be affected during FGR pregnancies since the exposure of these cells to serum from pregnant mothers with FGR has reported increased intracellular ROS generation and decreased viability, together with alterations in the mitochondrial structure, with damaged cristae, enlarged intramembrane space, and organelle swelling. The similarity of these findings with those observed in PE suggests a common course in the development of both disorders [57].

**Figure 3:**
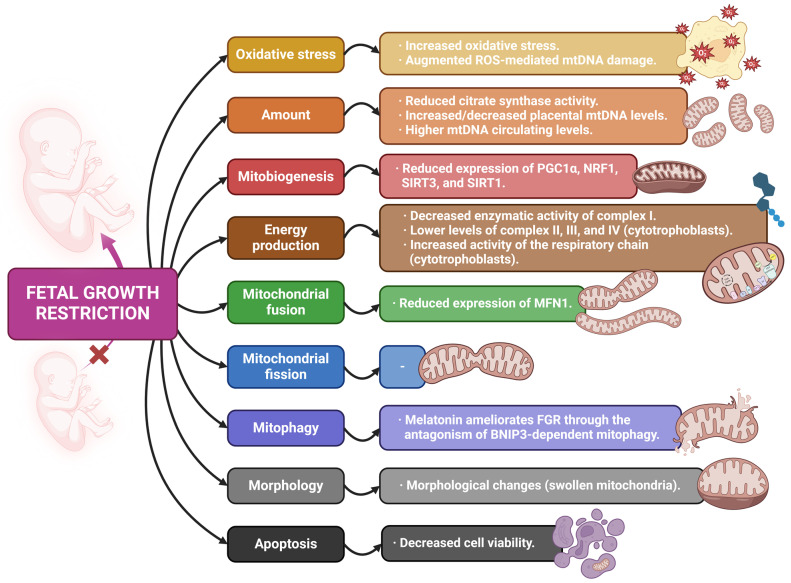
Summary of the mitochondrial alterations associated with fetal growth restriction found in the reviewed articles.

Finally, there is also a studied relationship between FGR and steroidogenesis, a process in which mitochondrial activity is fundamental. This pregnancy complication is highly dependent on proper levels of steroid hormones, associating hormonal disturbances with a higher risk of dysfunctional fetal growth [80]. Furthermore, aberrant methylation of the CYP11A1 gene, whose protein is essential for the synthesis of steroid hormones and is located in the IMM, has been found in the placentas of FGR cases [81]. Table 2 and Figure 3 contain a summary of the main findings derived from the reviewed articles related to FGR.

### 4.3. Mitochondria and Gestational Diabetes Mellitus

Gestational diabetes mellitus (GDM) is a heterogeneous condition depending on a combination of both genetic and environmental risk factors resulting in the impairment of insulin secretion and sensitivity, and its pathophysiology is quite similar to type 2 diabetes mellitus (T2DM) in most cases. In this sense, pancreatic beta cells are not able to adapt to increased insulin resistance, leading to high circulating glucose concentrations [82]. Furthermore, augmented adipose deposition, higher caloric intake, and little exercise cause a high body mass index (BMI) and contribute to insulin resistance. Pregnant women with this condition usually show a higher risk of hypertensive disorders, excessive fetal growth, and preterm complicated labor. GDM is a chronic low-grade inflammatory condition that can prolong insulin resistance after delivery, leading to the development of T2DM in mothers [83]. Long-term detrimental effects of GDM for mothers also include increased risk of cardiovascular diseases. The prevalence of this disease varies from 1–20%, and the proportion is rising worldwide together with the increase in the prevalence of obesity and T2DM [84].

Oxidative stress is highly associated with a hyperglycemic environment, which means that GDM pregnancies are characterized by both ROS overproduction and antioxidant mechanisms impairment (especially catalase), making the fetus more prone to be exposed to oxidative damage [85,86]. Higher lipid peroxidation and oxidized proteins, together with a lower antioxidant capacity, have been observed in GDM placentas [87], which has been suggested as possible causes of the increased incidence of malformations reported in newborns from diabetic women [88]. It should be highlighted that lipid peroxidation seems to limit mitochondrial respiration and ATP production in GDM pregnancies [89]. On the other hand, placentas from GDM pregnancies also show increased generation of the oxidized DNA product 8-OHdG, which is likely to affect mtDNA as well [90].

With regard to the antioxidant systems, placentas from GDM pregnancies have reported either downregulation due to increased ROS levels or upregulation as a compensation mechanism, dependent on the developmental stage and the gradual rise in ROS production (more pronounced at term) [91,92]. Some research has reported that hyperglycemic intrauterine environments lead to mtDNA permanent modifications that result in impaired mitochondrial biogenesis and activity, even after birth, possibly due to excessive oxidative stress [93]. Furthermore, thioredoxin-interacting protein (TXNIP), which promotes oxidative stress through the inhibition of thioredoxin (TXN), has reported increased protein expression in placentas from women with GDM [89] even though its gene expression has also shown to be reduced in GDM placentas [94]. However, this last study found elevated gene expression of TXNIP in maternal serum from GDM pregnancies [94]. This increase would lead to ROS accumulation, mitochondrial defects, apoptosis, and migration inhibition in trophoblast cells [89]. When it comes to peroxiredoxin 3 (PRX3), an H_2_O_2_ scavenger within the organelle, its levels have been shown to be augmented in mother plasma during GDM pregnancies [95].

In placental tissue from GDM pregnancies, electron chain complexes reported a significantly reduced expression. PGC1α, TFAM, and PPAR-γ protein expression were also reduced in these placentas, suggesting a relevant affectation in mitochondrial biogenesis regulation and energy metabolism [93,96,97]. When observing GDM placental ultrastructure, mitochondria architecture was found to be significantly altered, reflected by a high amount of swollen or destroyed organelles [98]. Interestingly, some of these results appear to be dependent on offspring sex since PGC1α protein abundance has been reported lower in placentas from male fetuses compared to those from females [93]. Something similar has been observed regarding TFAM [93,99], supporting the idea that GDM might be associated with damaged mitochondrial biogenesis in placentas from male fetuses. Nevertheless, other relevant proteins related to mitochondrial biogenesis have reported increased levels in samples from GDM pregnancies [87].

GDM placentas have reported a higher mtDNA copy number, while citrate synthase activity has been shown to be lower, suggesting a reduced number of mitochondria in these samples [100]. The content of mtDNA in the placentas of pregnant women with GDM and obesity was shown to be comparable to that measured in regular pregnancies. However, their mitochondria reported abnormal morphology, matrix density loss, and disorganized and damaged cristae [101]. On the other hand, pre-gestational diabetes and obesity have been related to increased ROS generation, together with reduced mtDNA content and complexes I-III activity and lower ATP generation [102,103]. Furthermore, the mitochondrial function of trophoblasts from women with GDM and obesity gets compromised since it favors anaerobic energy production. This is shown through augmented mRNA and protein levels of lactate dehydrogenase, hexokinase 2, and phosphofructokinase 2, which are associated with glycolysis [96]. Mitochondrial fatty acid metabolism has also reported alterations regarding GDM pregnancies, with a 30% reduction in β-oxidation in spite of the maintenance of the total mitochondrial content in the placenta [2].

In general, mitochondrial respiration rate appears to be negatively affected during GDM [100]. Women with GDM have shown reduced placental expression of the electron transfer complexes III and IV in comparison to placentas from regular pregnancies, possibly leading to lower effectiveness from electron transport and, eventually, ATP generation. There is also a difference comparing samples from women with GDM controlled by medication and women with GDM controlled by diet, where the expression of these complexes is reduced when the disease is controlled by medication [96]. In some studies, GDM did not seem to modify mitochondrial ATPase activity in the placenta [87], while others reported reduced respiration levels, mainly related to complexes I and II [100]. Likewise, umbilical endothelial cells from GDM pregnancies reported an alkaline intracellular pH, which leads to lower H+ levels in the mitochondrial intermembrane space, thus negatively affecting ATP generation by the V complex [104,105]. In addition, these cells showed a lower proliferation rate, which is quite dependent on ATP levels within cells [106].

Additionally, mitochondrial dynamics also seem to be affected in GDM-related pregnancies, with an augmented expression of OPA1, together with a reduced expression of pDRP1. This was supported by electron transmission microscopy showing a higher proportion of elongated mitochondria. The rebalance of mitochondrial dynamics towards fusion may be a potential adaptive mechanism and compensatory response, trying to protect the organelles through a mixture of their content. Placental ceramide content was also lower in GDM, which may be indicative of less active mitophagy processes [107]. MFN1 and MFN2 protein expression has also been shown to be elevated in GDM placental samples compared to controls, supporting the previous findings highlighting augmented fusion processes [100]. It has been suggested that some of these modifications might be secondary to hyperinsulinemia, which is a frequent feature of GDM pregnancies, as insulin has been related to a dose-dependent relationship with mitochondrial fusion promotion in JEG-3 cells [107]. Then again, there are studies reporting results that contradict this observed increase of fusion events, like the one carried out by Kolac et al. [97], which found decreased gene expression and protein levels of MFN1, MFN2, and OPA1 both in pre-DM placentas and GDM placentas in comparison to healthy pregnancies. Proteins related to mitochondrial protein folding were also reported to be decreased in this study.

**Figure 4:**
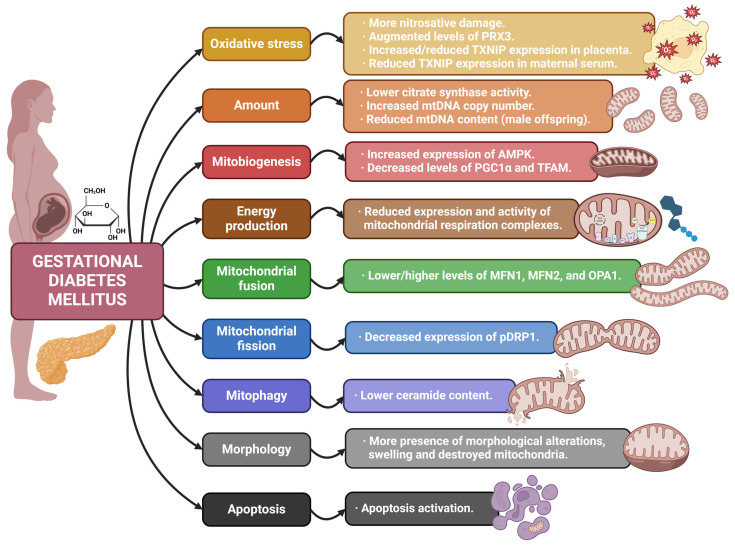
Summary of the mitochondrial alterations associated with gestational diabetes mellitus found in the reviewed articles.

Finally, the importance of steroid hormones in GDM must be pointed out too. Alterations in placental steroidogenesis and steroid hormone levels have been associated with increased insulin resistance and a higher risk of GDM [108]. Considering this, as well as the relevance of mitochondria in the proper synthesis of steroid hormones, the mitochondrial alterations observed during GDM could be translated into defects in steroidogenesis, thus affecting insulin resistance and favoring the appearance of this pregnancy disorder. However, this association still needs to be further explored and confirmed. Table 3 and Figure 4 summarize the main information of the reviewed articles on GDM.

The list of abbreviations used in this article is included in Table 4.

## 5. Conclusions

Mitochondria are key organelles in the proper function of highly active tissues and organs, including the placenta, since they are the powerhouses in charge of providing cells with energy and are also critical regulators of cell viability. Their activity can be altered by modifications in the oxygen supply derived from a reduction in placental blood flow, which usually is a common characteristic of pregnancy complications like those addressed in this review. Excessive ROS production and oxidative stress-related damage have been observed in the majority of studies concerning PE, FGR, and GDM, the mitochondria being the main source of free radicals. Likewise, several mitochondrial alterations have been documented in terms of content, structure, bioenergetics, dynamics, and adaptive responses, which point to mitochondria as important organelles to further understand these pregnancy disorders. However, these reported modifications vary from one pathology to another and even within the same disease. Timing, severity, gestational age, and the type of trophoblast cell have been proposed as possible explanations for the contradictory results obtained by different studies, sometimes proposing them as alterations in the mitochondrial machinery and sometimes as possible compensatory responses to mitochondrial impairment. Even though the underlying pathophysiology of pregnancy complications might differ, mitochondrial alterations can be a possible common feature among them, and as such, a proper understanding of the subject might offer opportunities for the development and testing of therapeutic interventions aimed at ameliorating pregnancy diseases. Nevertheless, further investigation is required so as to fully understand the complexity of the mechanisms regulating mitochondrial responses in these situations.

## Figures and Tables

**Table 1:** Summary of the major findings in articles about preeclampsia.

Reference	Disease	Study Design	Major Findings
[32]	PE	In vitro	Human umbilical vein endothelial cells exposed to plasma from women with PE report a decrease in mitochondrial function, as well as augmented mitochondrial superoxide production. The pre-treatment of cells with the mitochondrial-targeted antioxidant MitoTempo protects them against cell death induced by peroxide, also normalizing mitochondrial metabolism and reducing mitochondrial superoxide production.
[33]	PE	Observational Animal (mouse)	Placentas from subjects with PE show significantly high oxidative stress, mitochondrial damage, and reduced GPX activity. MitoQ late administration ameliorates uterine perfusion pressure, while early administration proves to be detrimental.
[34]	PE	Observational	OPA1 gene and protein expression is increased in early-onset PE placentas. TFAM expression is downregulated in comparison to controls. mtDNA copy number in early-onset PE placentas is significantly higher compared to normal pregnancies. Increase in citrate synthase activity is found in both early- and late-onset PE.
[37]	PE	Observational	PE placentas show reduced OXPHOS capacity (with no changes in gene or protein expression). Mitochondrial unfolded protein response (mtUPR) may be associated with these results, possibly being a therapeutic target for placental function restoration.
[38]	PE	Observational	Expression of mitochondrial fission/fusion markers, apoptosis proteins, and OXPHOS complexes are altered in a different way in term and pre-term PE placental samples. PE term placentas report increased expression of fusion markers and proteins related to apoptosis suppression. Mitochondrial content and respiration rate are elevated in term PE placentas.
[40]	PE	Observational	There is an abnormal regulation of mitochondrial dynamics, autophagy, and biogenesis in PE placentas, with reduced protein expression of fusion markers (OPA1, MFN2, and MFN1), the mitophagy receptor BNIP3, as well as mitobiogenesis mediators (PGC1α and SIRT3). Compromised lipid metabolism in these samples may result from dysfunctional mitochondria.
[45]	PE	Observational	Mitochondrial content is lower in placentas from PE mothers, with an increase in glycolysis components. Gene and protein expression of mitochondrial biogenesis regulators is lower in PE placentas, whereas the abundance of mitophagy-related proteins like BNIP3 is higher. Apoptosis activation and inflammation are also reported in placentas from PE women.
[46]	PE FGR	Observational	Gene expression of mitochondrial dynamics markers experiment changes in those placentas affected by PE and FGR. The TOMM20/Parkin ratio arises as a potential marker to discern unhealthy placental tissue.
[47]	PE	Observational In vitro	MFN2 gene and protein expression, as well as ATP levels, are significantly decreased in placentas from PE pregnancies compared to regular ones. MFN2 expression and TEV-1 cells’ viability are reduced during hypoxia. TEV-1 cells’ viability and ATP levels are also reduced after MFN2 knockdown. Consequently, defects related to MFN2 could cause mitochondrial alterations and negatively affect trophoblastic cells’ viability in PE.
[48]	PE	Observational In vitro	Mitochondrial dynamics in PE placental tissue are tilted toward fission (augmented DRP1 and reduced OPA1 expression). Transmission electron microscopy shows increased fragmentation of the organelle in PE placentas. Ceramides are more accumulated in the mitochondria from PE placentas. Mitophagy markers PINK1 and Parkin report an increase in placentas from PE pregnancies.
[49]	PE	Observational	The mitophagy receptor BNIP3 shows reduced expression in PE placentas, together with altered autophagy and augmented mitochondrial damage.
[50]	PE	Observational	Decidual gene transcription is modified in severe PE. Several genes related to glycolysis/gluconeogenesis, HIF-1 signaling pathway, and mitophagy (BNIP3) are importantly downregulated in the decidua of PE placentas.
[52]	PE	In vitro Animal (mouse)	Reduced gene expression and low ubiquitination of FUNDC1 are found in hypoxic trophoblast cells of pregnant women with PE.
[53]	PE	Animal (mouse)	Mitochondria swelling and cristae disappearance are observed in the trophoblasts of experimental PE groups.
[57]	FGR PE	In vitro	Endothelial cells’ short exposition with serum from pregnant mothers with FGR and PE slightly reduces cell viability. Prolonged exposition leads to important morphological changes (swollen mitochondria), increased ROS generation, autophagy, decreased cell viability.

**Table 2:** Summary of the major findings in articles about fetal growth restriction.

Reference	Disease	Study Design	Major Findings
[46]	PE FGR	Observational	Gene expression of mitochondrial dynamics markers changes in placentas affected by PE and FGR. The TOMM20/Parkin ratio arises as a potential marker to discern unhealthy placental tissue.
[57]	FGR PE	In vitro	Endothelial cells’ short exposition with serum from pregnant mothers with FGR and PE slightly reduces cell viability. Prolonged exposition leads to important morphological changes (swollen mitochondria), increased ROS generation, autophagy, decreased cell viability.
[63]	FGR	Observational	Lower mRNA levels of complex II, III, and IV are found in cytotrophoblast cells from FGR samples, without differences at the protein level. mtDNA is increased in FGR placentas, while mtDNA and NRF1 expression are significantly lower in isolated cytotrophoblast cells. The activity of cytotrophoblast respiratory chain is importantly augmented in placentas of FGR pregnancies.
[65]	FGR	Observational	mtDNA copy number is increased in FGR placental samples. Higher mutation rate is found in both coding and non-coding regions of mtDNA in several FGR placentas. SIRT3 expression is downregulated in FGR placentas.
[66]	FGR	Observational	mtDNA circulating levels are significantly higher in blood from mothers carrying FGR fetuses. SIRT3 expression is reduced in FGR placenta. Increased oxidative stress causes mtDNA damage in FGR.
[67]	FGR	Observational	The glycolysis-regulatory gene PDK1 is positively related to FGR. mtDNA content and oxidative stress are positively related to FGR.
[73]	FGR	Observational	FGR placentas show lower mtDNA and protein content (related to downregulation of ERRγ expression). Placental mtDNA content is directly correlated with fetal weight. PGC1α and SIRT1 gene expression is reduced in FGR+PE placentas.
[75]	FGR	Observational	mtDNA copy number in maternal venous blood is inversely associated with children’s birth weight, mostly in the third trimester. This might be a marker for identifying possible FGR.
[68]	FGR	Observational	FGR placentas report an important reduction in OXPHOS complex I enzymatic activity, together with complex I-stimulated oxygen consumption. They also show an increase in SIRT3 protein concentrations. Citrate synthase activity is significantly decreased in FGR newborns.
[77]	FGR	Observational	MFN1 protein expression is reduced in FGR placentas. TOMM20 gene and protein expression is increased in FGR placental tissue.
[78]	FGR	Animal (mouse)	Melatonin ameliorates Cd-caused FGR through the antagonism of BNIP3-dependent mitophagy in placental tissue, as well as the excessive release of ROS.

**Table 3:** Summary of the major findings in articles about gestational diabetes mellitus.

Reference	Disease	Study Design	Major Findings
[87]	GDM	Observational	Placentas with GDM are more susceptible to nitrosative damage as compared to normal placentas. GDM placentas report increased expression of AMPK, which may be associated with the maintenance of mitobiogenesis at a normal rate.
[89]	GDM	Observational In vitro	TXNIP expression in GDM placental tissue is increased compared to control. TXNIP expression in HTR-8/SVneo cells treated with high glucose is augmented, leading to ROS accumulation, mitochondrial defects, apoptosis, and migration inhibition.
[93]	GDM	Observational	Placental mitochondrial biogenesis is affected by GDM and offspring sex. GDM is associated with a reduction in PGC1α and TFAM levels in the placenta. Pregnancies with GDM and male offspring are related to reduced placental PGC1α and TFAM, as well as mtDNA content. Regarding female offspring, only decreased PGC1α is reported.
[94]	GDM	Observational	TXNIP gene expression is significantly elevated in serum of women with GDM. TXNIP gene expression is decreased in GDM placental tissue and cord blood. Pro-inflammatory alterations are related to low mRNA TXN/TXNIP ratio in maternal serum of GDM women but not in the placenta or in umbilical cord blood.
[95]	GDM	Observational	The H_2_O_2_ scavenger PRX3, with mitochondrial location, is increased in maternal plasma during pregnancies with GDM. It has an active role in the response to insulin release, suggesting it may be an indicator of high insulin resistance.
[96]	GDM	Observational	The expression of mitochondrial respiration complexes and PGC1α is significantly reduced in the placenta of women with GDM controlled by medication compared with women with GDM controlled by diet and controls.
[97]	GDM	Observational	Gene and protein expression fusion markers MFN1, MFN2, and OPA1 are lower in pre-DM and GDM placentas compared to healthy controls. PGC1α expression is reduced in these placentas as well. Proteins related to mitochondrial protein folding are also decreased in them.
[98]	GDM	Observational	GDM placentas show more swollen or destroyed mitochondria than those from regular pregnancies.
[101]	GDM	Observational	Obese women without GDM show increased mtDNA placental levels compared to normal weight women, suggesting increased biogenesis. The morphology of their mitochondria is similar. In obese women with GDM, mtDNA was not augmented compared to normal weight women, whereas morphological alterations are documented in their mitochondria.
[102]	GDM	Observational	Women with pre-existing maternal type 2 diabetes mellitus and obesity show elevated ROS production, decreased mtDNA content, reduced OXPHOS complexes I, II-III activities in placenta.
[100]	GDM	Observational	mtDNA copy number is higher in GDM placentas. Citrate synthase activity is shown to be lower in GDM placental samples. Placentas from GDM pregnancies report reduced respiration levels, mainly related to complexes I and II. Fusion proteins MFN1 and MFN2 report significant rises in GDM placentas.
[107]	GDM	Observational In vitro	Mitochondrial dynamics is tilted towards fusion in placentas of GDM women, supported by transmission electron microscopy and alterations in OPA1 (increases) and pDRP1 (decreased) expression. Placental ceramide content is lower in GDM. In vitro experiments show that increased insulin exposure promotes mitochondrial fusion.

**Table 4:** List of abbreviations used in the article in order of appearance.

Full Term	Abbreviation
Mitochondrial DNA	mtDNA
Reactive oxygen species	ROS
Preeclampsia	PE
Fetal growth restriction	FGR
Gestational diabetes mellitus	GDM
Oxidative phosphorylation	OXPHOS
Outer mitochondrial membrane	OMM
Inner mitochondrial membrane	IMM
Superoxide dismutase	SOD
Peroxisome proliferator-activated receptor-γ coactivator-1α	PGC1α
Nuclear respiratory factor 1	NRF1
Nuclear respiratory factor 2	NRF2
Mitochondrial transcription factor A	TFAM
Mitofusin 1	MFN1
Mitofusin 2	MFN2
Optic atrophy 1 protein	OPA1
Dynamin-related protein 1	DRP1
Dynamin 2	DNM2
Mitochondrial fission 1 protein	FIS1
PTEN-induced kinase 1	PINK1
E3 ubiquitin-protein ligase parkin	Parkin
Calcium binding and coiled-coil domain 2	CALCOCO2/NDP52
Optineurin	OPTN
FUN14 domain containing 1	FUNDC1
BCL2 interacting protein 3	BNIP3
BCL2-like 13 apoptosis facilitator	BCL2L13
FKBP prolyl isomerase 8	FKBP8
Prohibitin 2	PHB2
Mitochondrial permeability transition pores	mPTP
Soluble Flt-1	sFlt-1
Soluble endoglin	sENG
Natural killer	NK
Catalase	CAT
Glutathione peroxidase	GPX
Mitochondrial unfolded protein response	mtUPR
Sirtuin 3	SIRT3
Mitochondrial import receptor subunit TOM20 homolog	TOMM20
20α-dihydroprogesterone	20α-DHP
Intrauterine growth restriction IUGR	IUGR
Low birth weight	LBW
Estrogen-related receptor γ	ERRγ
Sirtuin 1	SIRT1
Type 2 diabetes mellitus	T2DM
Body mass index	BMI
Thioredoxin-interacting protein	TXNIP
Thioredoxin	TXN
Peroxiredoxin 3	PRX3
